# The Provider’s Experience of Delivering an Education-Based Wellness Program via Telehealth

**DOI:** 10.5195/IJT.2018.6268

**Published:** 2018-12-11

**Authors:** KATRINA M. SERWE

**Affiliations:** 1 DEPARTMENT OF OCCUPATIONAL THERAPY, SCHOOL OF HEALTH PROFESSIONS, CONCORDIA UNIVERSITY, MEQUON, WISCONSIN, USA

**Keywords:** Health care providers, Health personnel, Telehealth, Telehealth Usability Questionnaire (TUQ)

## Abstract

Provider acceptance is a first step to implementing a successful telehealth program. This pilot study examined the experience of six providers delivering an education-based wellness program in a telehealth format. Providers indicated an overall positive experience with high Telehealth Usability Questionnaire (TUQ) total scores (5.6 ± 1.1) and in their comments. High TUQ subscale scores for Usefulness (6.7 ± 0.4) and Ease of Use (5.3 ± 0.3) indicated providers found the telehealth system usable. Strong relationship bonds that developed offset the reported drawback of technical issues related to connectivity and audio. Providers with a wide range of computer experience all reported synchronous remote training via phone and videoconference meetings was adequate to prepare them to deliver classes via telehealth. This research indicates motivated providers with varying technology experience can have a positive telehealth experience with customized remote support.

Healthcare needs are changing, and healthcare costs continue to rise (Centers for Medicare & Medicaid Services, 2018). Disparities in health are increasing (Dickman, Himmelstein, & Woolhandler, 2017), and healthcare systems need innovative methods to reach populations who face health inequalities. The Triple Aim of Healthcare introduced the idea of an increased focus on population health management as a method to control costs while improving the experience of care and health (Berwick, Nolan, & Whittington, 2008). The goal is to promote equitable access to healthcare for all, with the promotion of access to care for populations at risk such as family caregivers, a growing population. An estimated 43.5 million Americans serve as an unpaid caregiver over course of a year (National Alliance for Caregiving & AARP Public Policy Institute, 2015).

Caregivers face barriers to accessing healthcare related to time, travel, availability of services, and health or caregiving demands that limit the ability to leave home (National Alliance for Caregiving & AARP Public Policy Institute, 2015). Furthermore, caregivers are at increased risk for both physical and mental health problems (Burton, Zdaniuk, Schulz, Jackson, & Hirsch, 2003; National Alliance for Caregiving & AARP Public Policy Institute, 2015). Caregivers are an asset to the healthcare system, as they reduce overall healthcare costs. In 2013, unpaid care was estimated at 470 billion dollars a year in the United States (Reinhard, Feinberg, Choula, & Houser, 2015). Increasing caregiver access to preventative services may decrease the health risks this population faces, as well as preserve a valuable asset to the healthcare system.

Telehealth offers a solution to many of the barriers caregivers report to accessing care. Telehealth is “the application of evaluative, consultative, preventative, and therapeutic services delivered through telecommunication and information technologies,” (American Occupational Therapy Association, 2013, p. 1). Telehealth allows access to services regardless of physical location, availability of transportation, and the availability of respite care. Telehealth also reduces travel-related costs for both providers and clients (Cohn, Brannon, & Cason, 2011). However, there are barriers to implementing telehealth, and a significant barrier may be provider acceptance (Mahoney, Tarlow, Jones, Tennstedt, & Kasten, 2001).

## LITERATURE REVIEW

Providers in the context of this research encompass any professional providing healthcare services such as physicians, nurses, social workers, physical therapists, occupational therapists, and others. Provider acceptance is a critical factor to telehealth adoption (Wade, Eliott, & Hiller, 2014). Providers must shift their roles and work habits to successfully adopt telehealth delivery methods (Segar, Rogers, Salisbury, & Thomas, 2013). Furthermore, providers tend to have a less positive view of telehealth than clients (Mair et al., 2005) and report a variety of concerns related to the adoption of telehealth delivery methods. These concerns include technical difficulties (Collier, Morgan, Swetenham, Currow, & Tieman, 2016; Levy & Neil, 2013); technology that is either inadequate or too expensive (Wade et al., 2014); lack of resources and organization support, including lack of technical support (Odeh, Kayyali, Nabhani-Gebara, & Philip, 2014); missing nonverbal cues resulting in decreased rapport with clients (Levy & Neil, 2013); time lag impeding conversation flow (Brandon et al., 2015); internet connectivity issues resulting in decreased rapport with clients (Collier et al., 2016; Holland et al., 2014); lack of reliable internet service (Sinclair, Holloway, Riley, & Auret, 2013); concern of increased workload (Collier et al., 2016; Odeh et al., 2014); concern for client safety (Shulver, Killington, & Crotty, 2016); and concern for decreased quality of care (Levy & Neil, 2013).

Despite the number of concerns providers report, there are factors that lead to provider satisfaction with telehealth. A good relationship with an information technology support team (Carlisle & Warren, 2013), familiarity with telehealth software through repeated use (Holland et al., 2014), previous experience with telehealth (Shulver et al., 2016), a younger age and more recent training (Sinclair et al., 2013), a video aspect to the telehealth technology (Collier et al., 2016), and a clear vision that telehealth will provide valuable services to clients (Carlisle & Warren, 2013; Collier et al., 2016; Levy & Neil, 2013; Shulver et al., 2016) are all factors associated with provider satisfaction with telehealth.

Providers who decide to participate in service delivery via telehealth often indicate this decision is based on client need (Carlisle & Warren, 2013; Levy & Neil, 2013). An Australian survey of healthcare providers found rural providers expressed a strong interest in telehealth regardless of their experience with technology, with a goal to provide better client outcomes (Shulver et al., 2016). Furthermore, providers have reported saving travel time for themselves and their clients, more efficient work, and increased safety as benefits of telehealth (Collier et al., 2016).

## PURPOSE

There are a variety of factors that influence provider attitudes toward telehealth. For a successful telehealth program, it is important to understand the providers’ experience of the telehealth delivery format and their needs for support. This study aimed to understand the providers’ experience of delivering a specific education-based wellness program for caregivers, Powerful Tools for Caregivers (PTC), via telehealth to learn how to best train providers and promote provider acceptance.

Providers of the PTC program are called class leaders. PTC class leaders undergo a 15-hour, two-day certification training program. PTC class leaders include a variety of professionals such as occupational therapy practitioners, social workers, nurses, family development specialists, and others. All class leaders have either direct experience as a caregiver or experience working with caregivers and their families.

The PTC program is a scripted, evidence-based, six-week education-based wellness program for caregivers. Program outcomes include improved caregiver emotional well-being, self-care behaviours, self-efficacy, and use and knowledge of community services. Details of the program and outcome are described elsewhere (Boise, Congelton, & Shannon, 2005). PTC class leaders deliver the program in pairs to model concepts and facilitate discussion. Traditionally, the PTC program is an in-person program.

This study examined the experience of six PTC class leaders (three leader pairs) involved in a pilot study of delivering the PTC program via telehealth. This study had one primary research question: What was the PTC class leaders’ experience of delivering the PTC program via telehealth? There were two additional questions. Did class leaders find the telehealth system usable? Did class leaders receive adequate training to deliver the PTC program via telehealth?

## METHODOLOGY

Between August 2017 and March 2018, three pairs of PTC class leaders each delivered one six-week PTC program via telehealth. Class leaders were purposefully selected with the assistance of the PTC National Office. Class leaders were selected to represent a variety of geographical areas that expressed a need to help caregivers gain access to PTC classes. The class leaders had different levels of experience in using technology, but all were experienced in co-leading the 6-week PTC class series. Two of the class leaders were National PTC Office staff and were included so they would gain direct experience with the new delivery format.

Class leaders received printed information, a link to online instructional and program materials, one formal training session, and ongoing informal support as needed from the principal investigator (PI). Class leaders delivered the telehealth classes using VSee software for synchronous videoconferencing as described in a previous feasibility study (Serwe, Hersch, & Pancheri, 2017). They assisted participants with downloading VSee software to participate in the telehealth class prior to the program start. Class leaders completed a survey related to their experience the week after they finished PTC telehealth program delivery.

The PI created the survey questions used in this pilot study based on a review of the literature. In addition to these custom created questions, the survey also included a modified version of the Telehealth Usability Questionnaire (TUQ). The TUQ assesses the usability of a telehealth system and has established reliability and validity (Parmanto, Lewis, Graham, & Bertolet, 2016). Five experts reviewed the survey to establish face validity. Expert reviewers included a software engineer and four occupational therapists with experience related to caregivers and telehealth. All reviewers were familiar with the VSee software used by the class leaders in this study. The PI updated the survey with minor revisions in response to reviewer feedback. One of the occupational therapist reviewers examined the final survey. The survey contained open and close-ended items. The PI used Qualtrics® survey software to deliver the survey. Class leaders received an email with an electronic link to the survey the week after their last telehealth PTC class.

The University Institutional Review Board approved this research. Class leaders received an email that described the purpose of the research and indicated that completion of the survey was consent to participate in the study.

## RESULTS

Six certified PTC class leaders who delivered a PTC program via telehealth participated in the study. This was the first PTC program the class leaders had delivered via telehealth. The six class leaders completed the survey for a 100% response rate.

All class leaders were female, Caucasian, and had experience with caregiving. They ranged in age from 50 to 71 years, with a mean age of 58 ± 11 years. Class leaders had experience leading in-person PTC classes for an average of 9.8 ± 5.6 years, with a range of three to 20 years. Class leaders were from Nebraska, Minnesota, or Oregon. They led PTC classes in service areas including a mix of rural, small town, suburban, and urban settings.

Over half of class leaders (66.7%) had not used videoconferencing prior to leading the telehealth PTC class. Two class leaders (33.3%) had previous experience with videoconferencing. One had experience using applications such as Skype, Messenger, Go to Meeting, and SocialZing to participate in meetings, webinars, and online training. The other had experience with VSee for work purposes and Skype for personal communication. Half of the class leaders reported they enjoyed using technology, and half reported they used technology when it is necessary. No class leaders reported avoiding technology use.

### COMPUTER SYSTEM SET UP

Computer system set up varied among class leaders. Only one class leader needed to obtain additional hardware to provide the telehealth PTC program. This class leader had a desktop computer and needed to obtain an external camera with a microphone to enable videoconferencing capabilities. This class leader had assistance from her workplace informational technology (IT) support team to install this additional hardware. Table 1 describes the class leaders’ computer set up.

### TELEHEALTH SYSTEM USABILITY

The TUQ, embedded in the full survey, provided information on the usability of a telehealth system for service delivery. TUQ items are rated on a seven-point Likert scale, with a rating of one indicating disagree and a rating of seven indicating agree; higher ratings indicate better system usability (Parmanto et al., 2016). The instrument provides subscale scores for assessing usefulness, ease of use, effectiveness, reliability, and satisfaction. Table 2 displays TUQ item, subscale, and total scores.

The TUQ includes one open-ended item that asks class leaders to provide comments on the telehealth system. Most class leaders reported an overall positive experience. Class Leader 1 commented,

I was so excited to be able to offer this course virtually to caregivers that would never have been able to go to a class in-person. It was easy to use, and our caregivers grasped the technology quickly. We are going to continue with this group doing an on-line support group.

Two class leaders commented that at first they had some problems with audio. “There were some challenges with volume levels initially in the first class that seemed to be resolved in future classes” (Class Leader 5). “Easy to use, felt comfortable. As [a] leader, [I] would like to stay on speaker without having to mute when others are talking (to avoid feedback) - to better facilitate brainstorms” (Class Leader 6). Class Leader 3 identified issues related to class participants’ computer hardware that were challenging but did not have an impact on group dynamics.

We had some difficulty with one of the participants using an iPad. Also, one of the ladies who started using a tablet had difficulty and [instead] used her computer. The tablet didn’t have enough battery power to provide both audio and visual. She did use it plugged in but that was difficult also. I think all those with a computer or laptop did well. Our ladies really bonded. Good experience.

Class Leader 2 described value in the telehealth method but had some concerns related to dealing with technology problems.

It [VSee] provided a system I would not otherwise have had and I was able to provide the valuable PTC with this method. I was unable to ‘fix’ a problem when it arose; but that’s most likely because of my inadequacy, not the system. We had some difficulty with static and several times, received a word message [VSee chat message] on a couple of the participants that they were receiving technology difficulties.

### BENEFITS OF TELEHEALTH TO CLASS PARTICIPANTS

Class leaders identified benefits they perceived caregivers in their classes received from the telehealth delivered PTC class. The primary benefit was that caregivers could take the class at home. This reduced barriers to attending classes such as driving, need to hire respite care for their care receiver, and in-person classes not offered near the caregiver’s home. Class leaders reported a benefit of caregivers being able to learn in their own home, gain self-care skills, increase confidence, and connect with other caregivers. Class Leader 4 described the connection between caregivers in her class.

[A benefit for caregivers was] Meeting other people who are walking in similar shoes. One of the things I was concerned [about was] if the group would create the same bond as [in] our in-person classes. I was amazed at how bonded they are and how supportive they are of each other. I also wondered how much they would share and react in an on-line class versus an in-person class. Again, I was amazed at how much they shared and connected with each other.

A comment from Class Leader 3 describes some of the benefits related to the telehealth delivery format, “I think overall it was good. Everyone could be in their homes. One of the participants was in different places during the classes, so she was able to travel and still be a part of this project.”

### DRAWBACKS OF TELEHEALTH

Four class leaders identified technology issues related to connectivity and audio issues. Audio issues were addressed by having participants mute their microphone when not talking. Class Leader 2 mentioned the format decreased spontaneity, and Class Leader 5 described a limitation of not being able to break up into smaller groups of two for discussion and not being able to view DVD content in the same manner as in the in-person classes. At two locations, class leaders shared a computer screen. One class leader commented it was a challenge to have both leaders close enough together to both be captured by the camera to appear in the video screen.

### TELEHEALTH CLASS LEADER EXPERIENCE COMPARED TO IN-PERSON EXPERIENCE

Class leaders reported a mix of pros and cons when comparing their telehealth experience to in-person class experiences. Class Leader 1 reported she did not feel as confident or connected to the group in the telehealth format. Class Leader 2 thought there was more “opportunity for sharing and emotions” in the in-person format. Class Leader 3 described a missing aspect of socialization before and after class in the telehealth format, but found it was easier to stay on schedule and there were fewer distractions in the telehealth format. Class Leader 4 thought her telehealth group was more open and willing to share, and there was value in the travel time saved for both the class leader and the class participants. Class Leader 5 reported a time lag in brainstorming in the telehealth format. She indicated an appreciation that class participants were better able to care for their needs in the telehealth format. “I very much appreciated how caregivers were able to attend to their needs (personal- getting a drink, caregiving - helping the care receiver) during the class.” Class Leader 6 described it as a similar experience with some exceptions.

Similar experience overall but was not able to reflect what caregiver is saying during feedback or brainstorms, [it] was tricky due to needing to mute/un-mute mic [the microphone]. We found a work around. Also, did not have the lingering afterward chatting as we do in some in-person classes.

### TIME REQUIREMENTS

All class leaders reported that delivery of the telehealth PTC classes did not require more time. Class Leader 2 described time saved using the telehealth delivery format.

I believe it took less time! At many locations for an in-person class, a leader has to arrive early to be there to greet the first arriving, to set up technology, and discover where all amenities are AND to clean-up after the class!

Four class leaders described time needed to train themselves and participants in software use but reported the telehealth format did not require more time than required to set up and deliver an in-person class.

### TRAINING

All class leaders described the written directions and training they received as “extremely adequate” to prepare for delivering PTC via telehealth. They shared advice related to the timing of training, stating it was easy to forget how to use aspects of the software if they did not start the telehealth class soon after their training. Class leaders also mentioned the importance of practicing with the software to feel comfortable before using in the telehealth classes.

## DISCUSSION

### CLASS LEADERS’ EXPERIENCE

The primary research question examined the class leaders’ experience of delivering the PTC program via telehealth. Class leaders indicated an overall positive telehealth experience as indicated by high TUQ ratings, positive comments, and positive responses to additional questions related to benefits, drawbacks, comparison of in-person to telehealth delivery, and time requirements. This is surprising given the majority of class leaders in this study had not used videoconferencing prior to leading the telehealth PTC class. Experience with technology is associated with a more positive telehealth experience (Shulver et al., 2016; Sinclair et al., 2013). However, this was not the case in this study. It is possible the class leaders’ perceived need of telehealth to meet caregiver needs, and the nature of the PTC course influenced their telehealth ratings.

Shulver, Killington, and Crotty (2016) found providers who believed there was a need for telehealth service delivery expressed a strong interest in telehealth despite their previous experience with technology. Class leaders in this study identified access for caregivers who would not otherwise be able to attend a PTC class as a major benefit of telehealth. Furthermore, telehealth delivered programs with a focus on teaching self-management skills have had high levels of provider satisfaction (Brandon et al., 2015; Carlisle & Warren, 2013) and are more likely to be sustainable (Radhakrishnan, Xie, & Jacelon, 2016). The PTC program teaches caregivers “tools” for self-management of stress, health, and caregiving responsibilities.

The drawback of technology issues, internet connection interruptions, and decreased spontaneity in conversation found in this study are consistent with previous research (Collier et al., 2016; Holland et al., 2014; Levy & Neil, 2013). However, despite impediments to communication, overall relationships may not be compromised. Holland et al. (2014) found that once a provider-client relationship was established, occasional poor video quality was “well tolerated” (p. 265). In this study, providers did not indicate video quality was the issue but rather audio quality. Occasional issues with audio quality were also well tolerated. Providers in this study described aspects of positive relationships such as the benefit of meeting people in similar situations and commenting on bonds formed between PTC class participants.

Five of the six providers indicated the nature of relationships changed from in-person to telehealth delivered classes. Four of the class leaders indicated there was less communication in the telehealth format. One reported the communication that did occur was more open as caregivers in their home setting shared personal information more freely. The differing nature of communication in the telehealth classes did not diminish the overall positive experience.

The previously reported provider concerns of increased time and workload related to telehealth service delivery (Collier et al., 2016; Odeh et al., 2014) were not realized in this study. No class leaders reported needing more time to deliver the telehealth PTC classes, and one class leader reported she saved time using the telehealth delivery format.

### USABILITY

The second research question examined usability. Did class leaders find the telehealth system usable? The TUQ was designed to assess the usability of telehealth implementation and services (Parmanto et al., 2016). The TUQ provides a total score and subscale scores for Usefulness, Ease of Use, Effectiveness, Reliability, and Satisfaction. The TUQ total score and all subscale scores were high, with means of 5.0 or higher for all. The subcategories of Usefulness and Ease of Use were the highest rated subscales. These high ratings indicate that class leaders did find the telehealth system usable.

### TRAINING AND SUPPORT

The final research question related to class leader training. Did class leaders receive adequate training to deliver the PTC program via telehealth? All class leaders rated the training they received with the highest rating, indicating their training was adequate. Previous research indicates tailored training is important for provider satisfaction of services delivered by telehealth (Brandon et al., 2015). Providers in this study received training with their partner class leader and individual training as requested. The PI provided one formal training session and additional sessions as needed along with printed and online materials. Training was done remotely by phone and using the telehealth software to videoconference. All class leaders reported this training was “extremely adequate” to prepare them to lead the telehealth classes.

In addition to initial training, ongoing support is important for a positive telehealth experience. Previous studies indicated support from IT professionals was a critical factor to provider satisfaction with telehealth, with a good relationship with IT professionals associated with satisfaction and lack of IT support a provider concern (Carlisle & Warren, 2013; Odeh et al., 2014). This study did not employ an IT support team. The PI served as the technical support person for training and questions related to the telehealth software. The PI had experience with the telehealth software but was not a trained IT professional. Some class leaders had local IT support available through their workplace, but only one class leader reported using outside support. This class leader requested workplace IT support to install a camera and microphone on her work computer in preparation for the telehealth program. She also requested that a friend with IT experience accompany her to assist with telehealth software installation on PTC class participants’ computers and training on software use. She recruited this assistance on her own. No other class leaders commented on outside support for computer or software related issues.

## LIMITATIONS

This study is limited by a small sample size and lack of diversity among class leaders. All six class leaders were female, of a similar age group, Caucasian, had shared history of experience with caregivers, and had a strong interest to share the PTC program with caregivers not able to attend the program in-person. Class leaders were selected from organizations that identified a need for telehealth services. Class leaders had a range of computer experience and comfort levels but were all motivated by a desire to meet needs in the communities they served. This level of motivation likely had a positive influence on their experience. Future research should involve a larger sample size of providers from diverse cultural, ethnic, gender, and age-groups.

## CONCLUSIONS

High TUQ ratings and class leader comments indicate an overall positive telehealth experience. Class leaders reported both benefits and drawbacks to the telehealth delivery method. The primary benefit class leaders noted was the opportunity for their PTC class participants to take the class at home and overcome many of the barriers related to attending in-person classes. Drawbacks of telehealth related to technical issues with connectivity and audio. Drawbacks were offset by strong relationship bonds that developed in classes. High TUQ scores indicated class leaders found the telehealth system and delivery format usable. Class leaders had a wide range of computer experience, but all reported their training was extremely adequate to prepare them to deliver the program via telehealth. Training occurred remotely via phone and videoconference meetings using the telehealth software. This research indicates a customized training program delivered remotely with ongoing support as needed may be adequate to prepare motivated healthcare providers to deliver an education-based wellness program via telehealth.

## Figures and Tables

**Table 1:** Class Leader Computer System Set Up

Computer System Attributes	Frequency (Percent)
Computer Style	
Laptop	4 (66.7%)
Desktop	2 (33.3%)
Operating System	
Windows	4 (66.7%)
Mac	2 (33.3%)
Internet Service	
Cable	3 (50.0%)
DSL (Digital Subscriber Line)	2 (33.3%)
Unknown	1 (16.7%)

**Table 2:** Telehealth Usability Questionnaire (TUQ) Results (n=6)

	Item	Mean (Standard Deviation)	Range
1.	Telehealth improves caregivers’ access to services, such as Powerful Tools for Caregivers.	7.0 (0.0)	7.0-7.0
2.	Telehealth saves me time traveling to get to classes.	6.8 (0.4)	6.0–7.0
3.	Telehealth met the participants’ needs to attend an educational program for caregivers.	6.3 (0.8)	5.0–7.0
	**Usefulness Scale Summary (Items 1–3)**	**6.7 (0.4)**	**6.3–7.0**

4.	It was simple to use this system.	5.0 (1.8)	2.0–7.0
5.	It was easy to learn this system.	5.0 (1.8)	2.0–7.0
6.	I was productive using this system.	5.8 (1.2)	4.0–7.0
7.	The way I interact with this system is pleasant.	5.5 (1.9)	2.0–7.0
8.	I like using this system.	5.2 (2.1)	2.0–7.0
9.	The system is simple and easy to understand.	5.3 (1.9)	2.0–7.0
	**Ease of Use Scale Summary (Items 4–9)**	**5.3 (0.3)**	**5.0 – 5.8**

10.	This system is able to do everything I would want it to be able to do.	4.7 (1.5)	2.0–6.0
11.	I can easily talk to others using the telehealth system.	5.7 (0.8)	5.0–7.0
12.	I can hear others clearly using the telehealth system.	5.7 (1.0)	4.0–7.0
13.	I felt I was able to express myself effectively.	5.5 (1.9)	2.0–7.0
14.	Using the telehealth system, I can see others as well as if we met in-person.	5.3 (1.2)	4.0–7.0
	**Effectiveness Scale Summary (Items 10–14)**	**5.4 (0.4)**	**4.7–5.7**

15.	I think the classes provided over telehealth are the same as in-person classes.	5.2 (1.0)	4.0–6.0
16.	Whenever I made a mistake using the system, I could recover easily and quickly.	4.8 (1.9)	2.0–7.0
17.	The system gave error messages that clearly told me how to fix problems.	5.0 (2.3)	1.0–7.0
	**Reliability Scale Summary (Items 15–17)**	**5.0 (0.2)**	**4.8–5.2**

18.	I feel comfortable communicating with others using the telehealth system.	5.8 (1.0)	5.0–7.0
19.	Telehealth is an acceptable way to deliver services.	6.2 (1.3)	4.0–7.0
20.	I would use telehealth to deliver a class again.	6.2 (1.3)	4.0–7.0
21.	Overall, I am satisfied with the telehealth system.	5.3 (2.1)	2.0–7.0
	**Satisfaction Scale Summary (Items 18–21)**	**5.9 (0.4)**	**5.3–6.2**

	TUQ Total Score	5.6 (1.1)	4.1 – 6.8

*Note*. Item 17 is the only item missing one response, as one class leader rated this as N/A.
